# *Trichinella spiralis* Calreticulin S-Domain Binds to Human Complement C1q to Interfere With C1q-Mediated Immune Functions

**DOI:** 10.3389/fimmu.2020.572326

**Published:** 2020-11-19

**Authors:** Shuai Shao, Chunyue Hao, Bin Zhan, Qinghui Zhuang, Limei Zhao, Yi Chen, Jingjing Huang, Xinping Zhu

**Affiliations:** ^1^ Department of Medical Microbiology and Parasitology, School of Basic Medical Sciences, Capital Medical University, Beijing, China; ^2^ Department of Pediatrics, National School of Tropical Medicine, Baylor College of Medicine, Houston, TX, United States

**Keywords:** *Trichinella spiralis*, calreticulin S-domain, binding site, complement C1q, classical complement activation, immune evasion, neutrophil, neutrophil extracellular traps

## Abstract

Helminths develop strategies to escape host immune responses that facilitate their survival in the hostile host immune environment. *Trichinella spiralis*, a tissue-dwelling nematode, has developed a sophisticated strategy to escape complement attack. Our previous study demonstrated that *T. spiralis* secretes calreticulin (*Ts*CRT) to inhibit host classical complement activation through binding to C1q; however, the C1q binding site in *Ts*CRT and the specific mechanism involved with complement-related immune evasion remains unknown. Using molecular docking modeling and fragment expression, we determined that *Ts*CRT-S, a 153-aa domain of *Ts*CRT, is responsible for C1q binding. Recombinant *Ts*CRT-S protein expressed in *Escherichia coli* had the same capacity to bind and inhibit human C1q-induced complement and neutrophil activation, as full-length *Ts*CRT. *Ts*CRT-S inhibited neutrophil reactive oxygen species and elastase release by binding to C1q and reduced neutrophil killing of newborn *T. spiralis* larvae. Binding of *Ts*CRT-S to C1q also inhibited formation of neutrophil extracellular traps (NETs), which are involved in autoimmune pathologies and have yet to be therapeutically targeted. These findings provide evidence that the *Ts*CRT-S fragment, rather than the full-length *Ts*CRT, is a potential target for vaccine or therapeutic development for trichinellosis, as well as for complement-related autoimmune disease therapies.

## Introduction

Trichinellosis, caused by the parasitic nematode, *Trichinella spiralis*, is a serious food-borne parasitic zoonosis affecting more than 11 million people worldwide (1). *T. spiralis* is a tissue-dwelling nematode with three life cycle stages: adult, newborn larvae (NBL), and infective muscle larvae (ML), which is encysted in host muscle tissue (2). During the life cycle, the parasite establishes orchestrated immune evasion strategies to avoid host immune attacks, from both the innate and adaptive immune systems.

The complement system is an important component of innate immunity that enhances the abilities of antibodies and phagocytic cells to clear microbes. Activated complement also directly attacks cell membranes and kills invading pathogens, including parasites (3). Over long-term evolution with their hosts, parasitic nematodes have developed sophisticated mechanisms to evade host complement attack. One mechanism used in this evasion is secretion of functional proteins that bind to different complement system components and interfere with their functions (4). *Ts*CRT is a calreticulin protein secreted by *T. spiralis* that has important roles in immunomodulation of the host immune system, including direct binding to the complement component, C1q (5), which is the initiator of the classical complement activation pathway, triggering a multistep activation cascade on binding to an immune complex (IC) (6). C1q is also involved in various cell processes by binding to its receptors on immune cells, such as macrophages, eosinophils, and neutrophils, to enhance their defensive abilities (7, 8). *T. spiralis* is a tissue-dwelling nematode, and its larvae invade intestinal epithelial cells and migrate to striated muscle, where they form an infective encysted ML. During this stage, the nematode is vulnerable to the host immune response, particularly complement attack, and *Ts*CRT is a multifunctional protein expressed by the nematode to bind to C1q and inactivate complement-mediated damage (5). Other parasites, like the intracellular protozoa *Trypanosoma cruzi*, filarial parasite *Brugia malayi*, hookworm *Necator americanus*, and nematode *Haemonchus contortus* also produce calreticulin to bind with C1q and inhibit classical complement activation on the parasite surface, thereby avoiding opsonization, immune stimulation, and lytic effects, as an immune evasion strategy (9–13).

In addition to initiating classic complement activation, C1q stimulates chemotaxis of neutrophils to the pathogen invaded region, and enhances their defense functions (14), which include production of reactive oxygen species (ROS) and neutrophil elastase (NE) (15, 16). C1q can also interact with a broad range of self and non-self ligands, such as IgG, IgM, the globular C1q receptor (gC1qR), *β*-amyloid peptide, lipopolysaccharide, and viral proteins, *via* its heterotrimeric globular head domain, which is composed of A (ghA), B (ghB), and C (ghC) chains (17). We previously demonstrated that *Ts*CRT can bind to host C1q; nevertheless, how the interaction of *Ts*CRT with C1q facilitates the survival of *T. spiralis* in the host under attack from complement and inflammatory cells, such as neutrophils, is not clear.

Calreticulin is a multifunctional protein with three distinct structural and functional domains: a globular N-domain, an extended proline-rich P-domain, and an acidic C-domain. The N and P domains are responsible for its chaperone function in the endoplasmic reticulum, while the C-domain contains a large number of negatively charged amino acids, responsible for high-capacity Ca^2+^ storage (18). A C1q-binding site has been located in the S-domain, which spans the intersection of the N and P domains of human calreticulin (19, 20). The tissue-dwelling filaria, *Brugia malayi*, secretes calreticulin (*Bm*CRT), that binds to host C1q through its N and P domains to inhibit the C1q-initiated classical pathway (11). Further, *Haemonchus contortus* calreticulin (*Hc*CRT) also uses its N domain to bind to C1q protein and restrict complement hemolytic activity (21). Nevertheless, the specific C1q binding site in *Ts*CRT has yet to be determined. Analysis and identification of the C1q binding site in the *Ts*CRT structure could further elucidate the mechanism involved in *T. spiralis* immune evasion and inform the development of strategies to enhance host immune responses to *Ts*CRT as a vaccine.

In addition to its functions in host defense, inappropriate activation or dysregulation of complement components can contribute to the development of autoimmune disease (22, 23). Neutrophil extracellular traps (NETs) are extracellular web-like structures, mainly comprised of DNA, histones, and granule proteins, such as elastase, and function to trap and kill invading pathogens, such as bacteria, fungi, viruses, and parasites (24, 25). IC-triggered C1q-initiated activation of the classical complement pathway can stimulate NET formation (NETosis) (26). In addition to the defense function of NETs, increasing evidence indicates that NETosis can also contribute to the pathogenesis of autoimmune disease (26, 27). Effective inhibitors of NETosis have been sought as potential therapeutic agents for autoimmune diseases (28, 29), and some complement modulators have been developed for the treatment of rheumatic diseases (30). Due to its strong C1q binding and inhibition functions, *Ts*CRT could be used to inhibit IC-C1q-initiated classical complement activation and NET formation and is, therefore, a potential therapeutic agent for autoimmune disorders, such as systemic lupus erythematosus, rheumatoid arthritis, psoriasis, and antiphospholipid syndrome (29).

In this study, the C1q binding site in *Ts*CRT was identified and shown to confer immunomodulatory functions, including inhibition of classical complement activation, C1q-mediated neutrophil functions, and NET formation. Determination of the C1q binding site in *Ts*CRT has potential to facilitate vaccine development against *T. spiralis* infection, as well as potential therapeutic targets for autoimmune diseases involving complement.

## Materials and Methods

### Experimental Animals

Female BALB/c mice aged 6–8 weeks were purchased from the Laboratory Animal Services Center of Capital Medical University (Beijing China) and maintained under specific pathogen-free conditions at 20 ± 2 °C; humidity, 60 ± 10%. All animal protocols were approved by Capital Medical University Animal Care and Use Committee (approval number: AEEI-2017-133) and complied with the NIH Guidelines for the Care and Use of Laboratory Animals.

### Sera

Normal human serum (NHS) was collected from the blood of healthy human volunteers, according to a protocol approved by the Institutional Review Board (IRB) of Capital Medical University (approval number: 2016SY01). Human C1q-deficient serum (C1q-D) was purchased from Merck (Darmstadt, Germany).

### Parasites

*T. spiralis* (strain ISS 533) was maintained in female mice, and NBL were collected from fertile female adult worms cultured in phenol red-free RPMI 1640 (Gibco by Life Technologies, Carlsbad, USA) with 1× penicillin–streptomycin (Solarbio, Beijing, China) at 37°C for 48 h.

### Molecular Docking

Molecular docking of *Ts*CRT with human complement C1q was performed using Discovery Studio 2017 software (Neotrident Technology Ltd., Beijing, China). The three-dimensional structure of *Ts*CRT was developed using the structure of calreticulin arm domains (PDB: 3RG0) as a template. The best *Ts*CRT structure was acquired, according to the DOPE score and PDF total energy. Alignment of the *Ts*CRT sequence (XP_003371379.1) with the template was conducted and a refined structure generated. Docking was conducted using ZDOCK and refined with RDOCK in Discovery Studio 2017.

### Expression of Recombinant *Ts*CRT and Its Fragment Proteins 

Based on the structure of calreticulin, full-length *Ts*CRT (*Ts*CRT, 22–413 amino acids (aa)) and its various domain fragments were expressed as recombinant proteins in *Escherichia coli*. The domain fragments included the NP-domain (22–331 aa), P-domain (*Ts*CRT-P, 172–331 aa), C-domain (*Ts*CRT-C, 332–413 aa), and S-domain, a potential C1q binding site spanning the N and P domains (*Ts*CRT-S, 135–288 aa) (**Figure 2A**). The DNA sequences encoding these fragments were subcloned into the pET-24a bacterial expression vector (Novagen, Darmstadt, Germany) using the specific primers listed in **Table 1**. Recombinant plasmids with confirmed correct sequences were transformed into *E. coli* BL21(DE3). Recombinant fragments with c-terminal His-tags (r*Ts*CRT, r*Ts*CRT-NP, r*Ts*CRT-P, r*Ts*CRT-S, and r*Ts*CRT-C) were expressed by induction using 1 mM IPTG at 37°C for 3.5 h, and then purified by Ni-affinity chromatography (Novagen, Madison, USA). Endotoxin contaminating the purified recombinant proteins was removed using Pierce High Capacity Endotoxin Removal Resin (Invitrogen, Carlsbad, CA, USA), and endotoxin removal was confirmed using the ToxinSensor Endotoxin Detection System (GenScript, Nanjing, China). The concentration of purified proteins was measured using a BCA Protein Assay Kit (Thermo Fisher, Carlsbad, USA).

**Table 1:** Primers used in expression of each fragment of *Ts*CRT.

	Forward	Reverse
***Ts*CRT**	CTAGCTAGCGAGCCGACCATTTACCTCAAG	GCCTCGAGTCAATGATGATGATGATGATGCAGCTCTTCTTTAACATTTTC
***Ts*CRT-NP**	CTAGCTAGCGAGCCGACCATTTACCTCAAG	GCCTCGAGTCAATGATGATGATGATGATGTTCAAAACCGATTGCCCCAAG
***Ts*CRT-P**	CTAGCTAGCACGCATGCGTACAAATTAATC	GCCTCGAGTCAATGATGATGATGATGATGTTCAAAACCGATTGCCCCAAG
***Ts*CRT-S**	CTAGCTAGCATGTTCGGACCTGATATATGTGGACCA	GCCTCGAGTCAATGATGATGATGATGATGATCAGGATTTGGAATTTGTTCCGGTGCCC
***Ts*CRT-C**	CTAGCTAGCCTGTATCGATACAAAGGT	GCCTCGAGTCAATGATGATGATGATGATGCAGCTCTTCTTTAACATTTTC

### Assays for *Ts*CRT and Its Human C1q Binding Fragments 

#### ELISA

To compare the binding affinity of *Ts*CRT and its fragments to C1q, 96-well plates were coated with different amounts of human C1q (0, 0.05, 0.1, 0.2, 0.4, 0.8, 1.2, and 1.5 µg/ml) (Millipore, Darmstadt, Germany) in 100 μl/well of coating buffer (100 mM Na_2_CO_3_/NaHCO_3_, pH 9.6) overnight at 4°C. The same amounts of BSA were coated in the control wells. Wells were then washed with PBS + 0.05% Tween-20 (PBST) three times, followed by blocking with 200 µl 3% BSA in PBS at 37°C for 1 h. After further washing, 100 µl of 40 nM r*Ts*CRT, r*Ts*CRT-NP, r*Ts*CRT-P, r*Ts*CRT-S, and r*Ts*CRT-C in binding buffer (100 µl of 20 mM Tris–HCl, pH 7.4, 50 mM NaCl, and 1 mM CaCl_2_) was added to each well and incubated for 1 h at room temperature. Mouse anti-His mAb (1:10,000, Tiangen, Beijing, China) and HRP-conjugated goat anti-mouse IgG (1:10,000, BD Biosciences, Franklin Lakes, USA) were used as primary and secondary antibodies, respectively. After washing with PBST, the substrate, o-phenylenediamine dihydrochloride (Sigma, St. Louis, MO, USA) was added; colorimetric detection was performed using TMB and the absorbance measured at 450 nm using an ELISA reader (Thermo Fisher).

#### Far Western Blotting

Human C1q (5 µg) and BSA (5 µg) were separated on 12% SDS-PAGE gels and transferred onto nitrocellulose membranes, which were blocked with 3% BSA and then incubated with 5 µg/ml of r*Ts*CRT, r*Ts*CRT-NP, r*Ts*CRT-P, r*Ts*CRT-S, and r*Ts*CRT-C in binding buffer containing 1 mM CaCl_2_ at 37°C for 2 h. Anti-His mAb (1:5,000) was used to detect each fragment bound to C1q, with IRDye 800CW-conjugated anti-mouse IgG (Li-COR, Lincoln, NE, USA) as the secondary antibody, followed by visualization using an Odyssey CLx Infrared Imaging System.

#### Immunoprecipitation

To determine whether Ca^2+^ was required for binding of *Ts*CRT-S to C1q, r*Ts*CRT-S (2 µg) was incubated with anti-His mAb (2 µg) and Protein G MicroBeads (Miltenyi Biotec, Cologne, Germany) in PBS (50 µl) with or without 1 mM CaCl_2_ for 30 min on ice. Human C1q (3 µg) was added into the bead complex and incubated overnight at 4°C. After washing in washing buffer (1% NP40, 50 mM Tris–HCl, 50 mM NaCl, pH 8.0), the binding complex on the beads was stripped with preheated loading buffer (1× SDS, 50 mM Tris–HCl) using a magnetic field. Then, the eluted protein complex was separated by SDS-PAGE and transferred onto a nitrocellulose membrane. Rabbit anti-human C1qA antibody (1:10,000) (Abcam, Cambridge, UK) was used to detect the C1q immunoprecipitated with r*Ts*CRT-S. Binding conditions without Ca^2+^ were used as a control.

### r*Ts*CRT-S Inhibits C1q-Induced Classical Complement Activation 

#### C4b and C3b Deposition Assay

C1q-induced classical complement activation was performed as previously described (5). Briefly, human IgM (2 µg/ml) (Millipore) was used to coat 96-well plates, which were incubated with 100 µl/well of C1q (1 µg) that had been pre-incubated with different amounts of r*Ts*CRT-S (1, 2, and 4 µg), r*Ts*CRT (2 µg), or BSA (2 µg) at 37°C for 1 h. The plates were washed with PBST and incubated with C1q-D (1:100) in GVBS^++^ buffer (1× Veronal buffer, Lonza, Switzerland), containing 0.1% gelatin, 0.15 mM CaCl_2_, and 1 mM MgCl_2_) for 1 h at 37°C. NHS (1:50) served as a positive control. Subsequently, mouse anti-human C4b mAb and rabbit anti-human C3b polyclonal antibodies (Abcam, Cambridge, UK) at dilution of 1:5,000, were used to probe the classical complement activation intermediate products, C4b and C3b, and HRP-conjugated goat anti-mouse or goat anti-rabbit IgG (1:10,000, BD Biosciences) used to detect the deposited C4b and C3b. Absorbance was read at 450 nm using an ELISA reader.

#### Hemolytic Assay

To determine the inhibition effect of r*Ts*CRT-S on C1q-initiated classical complement activation-mediated hemolysis, human IgM (2 µg/ml) was coated in 96-well plates. 1 µg of C1q was incubated with different amounts of r*Ts*CRT-S, (0.5, 1, 2, 4, and 6 µg), r*Ts*CRT (4 µg), or BSA (6 µg), followed by addition of C1q-D (1:50) in 1× HBSS^++^ (Hank’s balanced salt solution, Thermo Fisher, containing 1 mM MgCl_2_ and 0.15 mM CaCl_2_) for 1 h at 37°C. Fresh sheep red blood cells (SRBC, 5 × 10^6^ cells/ml in HBSS^++^) were sensitized using rabbit anti-SRBC antibody (Sigma, USA) at 37°C for 30 min, then added into the reaction complex with C1q-D for 30 min. Hemolysis was stopped using cold HBSS^++^ containing 10 mM EDTA. The mixture was then centrifuged at 1,500 × g for 10 min. Hemoglobin in the supernatant was measured at an absorbance of 412 nm. Hemolytic activity was calculated as a percentage of total hemolysis in water.

### Culture and Differentiation of HL60 Cells

The human promyelocyte cell line, HL60, carries many neutrophil receptors and can be differentiated into neutrophil-like cells for studies of neutrophil function (31). HL60 cells were obtained from ATCC and maintained in RPMI 1640 medium (Invitrogen) containing 10% fetal bovine serum (FBS, Gibco) and 1× penicillin–streptomycin. Cells were stimulated with 1 µM all-trans retinoic acid (ATRA) (Sigma) for 5 days to differentiate into neutrophil-like cells (dHL60) (32, 33). To confirm dHL60 cell differentiation, ATRA-induced HL60 cells were resuspended in FBS-free RPMI 1640 medium and allowed to settle for 20 min for differentiated dHL60 cells to adhere on round glass slides in a 24-well plate, followed by fixing in 4% paraformaldehyde for 40 min (34). Adhered cells were washed with PBS and stained with DAPI dye (Zhongshanjinqiao, Beijing, China) for observation of lobulated nuclei. Differentiation of dHL60 cells was also confirmed by evaluating expression of CD11b and CD66a *via* qPCR and flow cytometry.

### Neutrophil Isolation From Human Peripheral Blood

Human polymorphonuclear neutrophils (PMNs) were isolated from the peripheral blood of a healthy volunteer, using Ficoll-Paque PLUS (Solarbio) with a standard protocol. Briefly, whole fresh blood was collected in a heparin-coated tube, and PMNs were purified by density gradient centrifugation (1000 × g, 30 min) and hypotonic erythrocyte lysis on ice for 10 min. Cells were counted and suspended in RPMI 1640 medium containing 10% FBS.

### r*Ts*CRT-S Inhibits C1q-Induced dHL60 Activity 

#### C1q Binding Inhibition

To determine whether r*Ts*CRT-S or r*Ts*CRT inhibited C1q binding to C1q receptor on dHL60 cells, C1q (25 nM) was pre-incubated with different amounts of r*Ts*CRT-S (0, 0.5, 1, and 2 µM) or r*Ts*CRT (2 µM), then added to dHL60 cells that adhered on 96-well plates (5 × 10^4^/well) or coverslips (3 × 10^5^/well), as described above, for 1 h at 37°C. After washing, rat anti-C1q mAb (1:100, Abcam) was used to detect the binding of C1q to dHL60 cells at 4°C overnight, and DyLight 488-conjugated goat anti-rat IgG (1:100) (KPL, Milford, USA) was used as the secondary antibody. After staining, the mean fluorescence intensity (MFI) of C1q on cells in the 96-well plate was measured by High Content Analysis (Thermo Fisher). Cells containing C1q and nuclei staining were captured and analyzed by confocal laser scanning microscopy (Leica).

#### Transwell Chemotaxis Assay

To determine the effects of r*Ts*CRT-S and r*Ts*CRT on C1q-induced chemotactic migration of dHL60 cells, a Transwell chamber with 3-µm-pore membranes (Corning, NY, USA) was inserted in a 24-well plate. dHL60 cells (2 × 10^5^ per well) were added to the upper chamber in 1640 medium with 5% FBS. The lower chamber was filled with C1q (50 nM) pre-incubated with different amounts of r*Ts*CRT-S or r*Ts*CRT (50 and 100 nM) in FBS-free medium to induce chemotactic migration at 37°C in a 5% CO_2_ incubator for 6 h. After washing, cells that had migrated through the membrane into the lower chamber were counted using a Celigo^®^ Image Cytometer (Nexcelom, Lawrence, MA, USA). BSA or recombinant proteins (100 nM) without C1q were used as negative controls.

#### Measurement of Reactive Oxygen Species

To measure the effect of *Ts*CRT on C1q-induced neutrophil activity, ROS produced by activated neutrophils was detected with 2′,7′-dichlorofluorescin diacetate (DCFH-DA, Sigma). C1q was coated in 96-well plates (1 µg/well) at 4°C overnight, then incubated with different amounts of r*Ts*CRT-S or r*Ts*CRT (0, 0.5, 1, and 2 µM) in binding buffer at 37°C for 1 h. BSA (2 µM) was used as a control protein. PMA (160 nM) was used as a positive stimulation control. The ROS inhibitor, cysteamine (5 mM), was used as a negative control. A total of 5 × 10^4^ dHL60 cells were added in each well. After being incubated at 37°C in a 5% CO_2_ incubator overnight, ROS activity from HL60 cells was detected using 10 µM DCFH-DA. Hoechst 33342 was used to stain nuclei in living cells. ROS content was quantified as MFI using High Content Analysis.

#### Measurement of Neutrophil Elastase

dHL60 cells were stimulated with C1q pre-incubated with different amounts of r*Ts*CRT-S or r*Ts*CRT in 96-well plates, as described above. The NE activity of C1q-stimulated dHL60 cells was measured using a NE activity kit (Abcam) on a ﬂuorescence microplate reader (FLx800, BioTek) at 380/500 nm. The elastase inhibitor, SPCK (60 µM), was used as a negative control.

#### C1q-Mediated Neutrophil Attack on NBL *In Vitro*

To determine whether r*Ts*CRT-S or r*Ts*CRT influences C1q-induced neutrophil killing on NBL, a CellTiter-Glo^®^ Luminescent Cell Viability Assay (Promega, Madison, WI, USA) was used to measure ATP levels in parasites, to determine NBL viability (35, 36). Briefly, C1q was coated on 24-well plates (3 µg/well) at 4°C overnight, followed by incubation with different amounts of r*Ts*CRT-S or r*Ts*CRT (0, 3, and 6 µM) and BSA (6 µM) in 300 µl binding buffer at 37°C for 60 min. Next, each well was filled with PMN isolated from a healthy donor (5 × 10^5^/500 µl), a transwell chamber added into each well, and a total of 500 NBL added to each upper chamber. After incubation at 37°C for 48 h, NBL in the upper chambers were collected and washed with phenol red-free 1640 medium. CellTiter-Glo^®^ Reagent was added, and ATP activity in the larvae was measured, according to the manufacturer’s instruction. The luminescence generated was measured using a ﬂuorescence microplate reader (FLx800, BioTek). ATP levels were calculated using a standard curve generated from the ATP levels in untreated control NBL.

### Neutrophil Extracellular Traps Assays

#### Quantitation of NET Formation

Cell-free extracellular DNA released by neutrophils is an important component of neutrophil extracellular traps (NETs). To determine the potential *Ts*CRT inhibition of complement-induced NETosis, through reduction of C1q function, DNA involved in net-like NET was digested using DNase I and free DNA measured using PicoGreen. IC was generated by incubating 10 µl anti-chicken egg albumin antibody (Sigma) with an equal volume of 0.25 mg/ml albumin from chicken egg (Sigma) at 37°C for 30 min. C1q (2 µg) was pre-incubated with different amounts of r*Ts*CRT-S, r*Ts*CRT (0, 0.1, 0.3, and 0.9 µM), or BSA (0.9 µM) in 50 µl binding buffer at 37°C for 60 min, followed by incubation with IC at 37°C for 30 min. Subsequently, mixtures were added to 300 µl GVBS^++^ buffer with 3% C1q-D and incubated at 37°C for 30 min, to activate the complement system. For NET formation induced by activated complement, this combination was added to dHL60 or PMN cells adhered in 96-well plates (10^4^/well). Cells were incubated for 6 h (dHL60) or 1 h 40 min (PMN) at 37°C in a 5% CO_2_ incubator. H_2_O_2_ (0.05%) was further added into dHL60 cells to enhance NET formation. DNase I (200 U/ml; Roche) was added to each well to digest released extracellular DNA for 10 min in a 37°C incubator. After centrifugation, supernatants containing digested DNA were transferred into a new 96-well plate and mixed 1:1 (v/v) with PicoGreen reagent (Thermo Fisher). Fluorescence was then quantified on a BioTek microplate reader at 485 nm/528 nm.

#### Observation of NET Formation by Fluorescence Confocal Microscopy

dHL60 cells or PMN were adhered on 14-mm round glass slides coated with PLL (Liangyi, Dalian, China) and stimulated using activated complement complex, as described above, then fixed overnight in 2.5% glutaraldehyde at 4°C. For staining, slides were washed three times in PBS and blocked with goat serum working buffer (Zhongshanjinqiao, Beijing, China) and 2% BSA in PBS for 1 h at room temperature (RT). Rabbit anti-elastase (1:300, Merck) and mouse anti-histone H3 (1:500, Abcam) were used as primary antibodies in PBS containing 2% BSA for 1 h at RT. After washing, slides were incubated with goat anti-rabbit DyLight 488 (KPL) and goat anti-mouse Alexa Fluor 594 (Thermo) at 1:1,000 for 1 h at room temperature. Slides were then stained with DAPI and visualized using a confocal laser scanning microscope camera (Leica).

#### Observation of NET Formation by Scanning Electron Microscopy

dHL60 and PMN cells, treated as described above, were analyzed by scanning electron microscopy (SEM) after being fixed with 2.5% glutaraldehyde and dehydrated with gradually increased concentrations of ethanol (50, 70, 80, 90, and 100%), then dried with a critical point dryer and coated (Leica). Cells were observed using a Hitachi S-4800 scanning electron microscope (Hitachi High-Tech Corp., Tokyo, Japan).

### Statistical Analysis

Results are presented as means ± standard error of the mean (SEM) and were analyzed by one-way analysis of variance using GraphPad Prism 7 software (San Diego, CA, USA); *p* < 0.05 was regarded as statistically significant.

## Results

### Predicted Structure and C1q Binding Sites of *Ts*CRT

**Figure 1:**
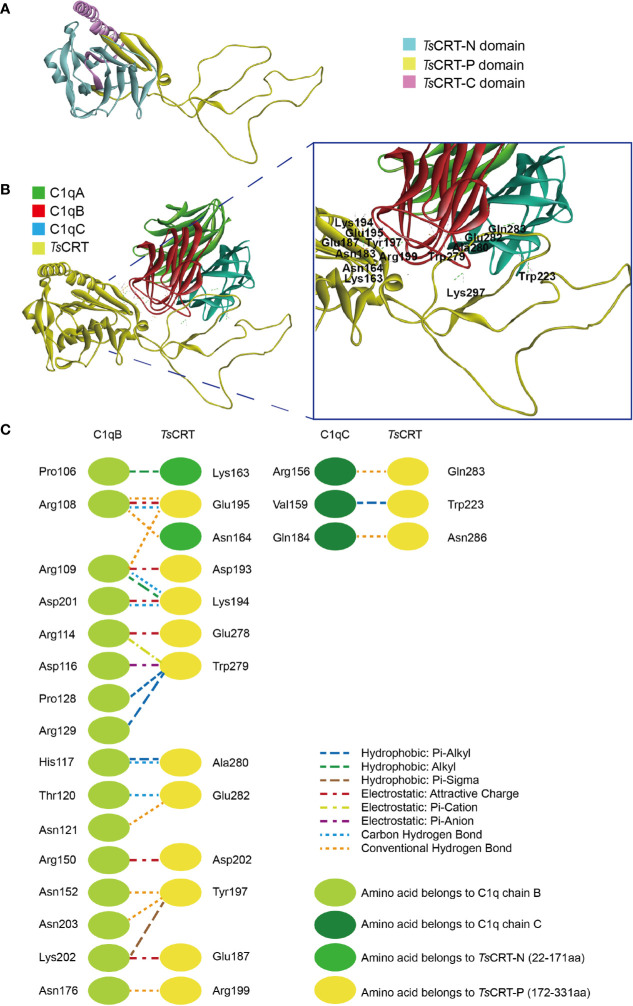
Predicted protein–protein docking of *Ts*CRT and human C1q (1PK6) model. **(A)** Modeled 3D structure of *Ts*CRT, showing the N domain (light blue), P domain (yellow), and C domain (pink). **(B)** Predicted C1q binding site in *Ts*CRT located in the N and P domains, showing that C1qB and C1qC of C1q interact with *Ts*CRT amino acids. **(C)** Interface analysis of the C1q-*Ts*CRT binding site, showing amino acids and their bond details.

Homology modeling of the *T. spiralis* calreticulin structure was established using the crystal structure of human calreticulin arm domains (PDB: 3RG0) as a template; the molecules share 47.6% sequence similarity. The 3D structure of *Ts*CRT with the lowest PDF total energy was chosen (**Figure 1A**). *In silico* analysis of the *Ts*CRT amino acid sequence demonstrated that the structure of *Ts*CRT is composed of a globular domain (N-terminal *β*-sheet and C-terminal *α*-helix) and a proline-rich loop (P-domain). Analysis of the protein–protein interaction between *Ts*CRT and C1q showed that residues located within the *Ts*CRT N and P domains were involved in binding with C1q chains B and C (**Figure 1B**). Specifically, amino acids Lys^163^ and Asn^164^, in the N-domain, and Glu^187^, Asp^193^, Lys^194^, Glu^195^, Tyr^197^, Arg^199^, Asp^202^, Glu^278^, Trp^279^, Ala^280^, and Glu^282^, in the P-domain interacted with Pro^B106^, Arg^B108^, Arg^B109^, Arg^B114^, Asp^B116^, His^B117^, Thr^B120^, Asn^B121^, Pro^B128^, Arg^B129^, Arg^B150^, Asn^B152^, Asn^B176^, Asp^B201^, Lys^B202^, and Asn^B203^ of the C1q B chain. Some amino acids Trp^223^, Gln^283^, and Asn^286^ in the P-domain are involved in interactions with Arg^C156^, Val^C159^, and Gln^C184^ in the C1q C chain (**Figure 1C**). The interaction between *Ts*CRT and C1q was mostly *via* hydrogen, electrostatic, and hydrophobic bonds. *In silico* docking predicted that C1q binding sites in *Ts*CRT are mainly located between Lys^163^ in the N-domain and Asn^286^ in the P-domain.

Amino acids in human C1q involved in C1q–IgG, C1q–IgM, C1q–gC1qR, and C1q–*Ts*CRT interactions were also evaluated (17, 37, 38) (**Table 2**). The docking model suggested that C1q Arg^B108^, Arg^B109^, Arg^B114^, His^B117^, and Arg^C156^, which were involved in interaction with C1q–*Ts*CRT, also contribute to the interactions with C1q–IgG, C1q–IgM, and C1q–gC1qR.

**Table 2:** Details of amino acids on human C1q that are involved in C1q–IgG, C1q–IgM, C1q–gC1qR and C1q–*Ts*CRT interactions.

	Amino acids	Reference
HuC1q-IgG	Arg^A162^, Arg^B108^, Arg^B109^, Arg^B114^, His^B117^, Arg^B129^, Arg^B163^, Tyr^B175^, and Arg^C156^	(17, 37)
HuC1q-IgM	Arg^B108^, Arg^B109^, and Tyr^B175^	(17)
HuC1q-gC1qR	Arg^B114^, His^B117^, Arg^B163^, and Arg^C156^	(38)
HuC1q-*Ts*CRT	Pro^B106^, Arg^B108^, Arg^B109^, Arg^B114^, Asp^B116^, His^B117^, Thr^B120^, Asn^B121^, Pro^B128^, Arg^B129^, Arg^B150^, Asn^B152^, Asn^B176^, Asp^B201^, Lys^B202^, Asn^B203^, Arg^C156^, Val^C159^, and Gln^C184^	Shown in this manuscript

### Expression and Characterization of Recombinant *Ts*CRT and Its Fragments

**Figure 2:**
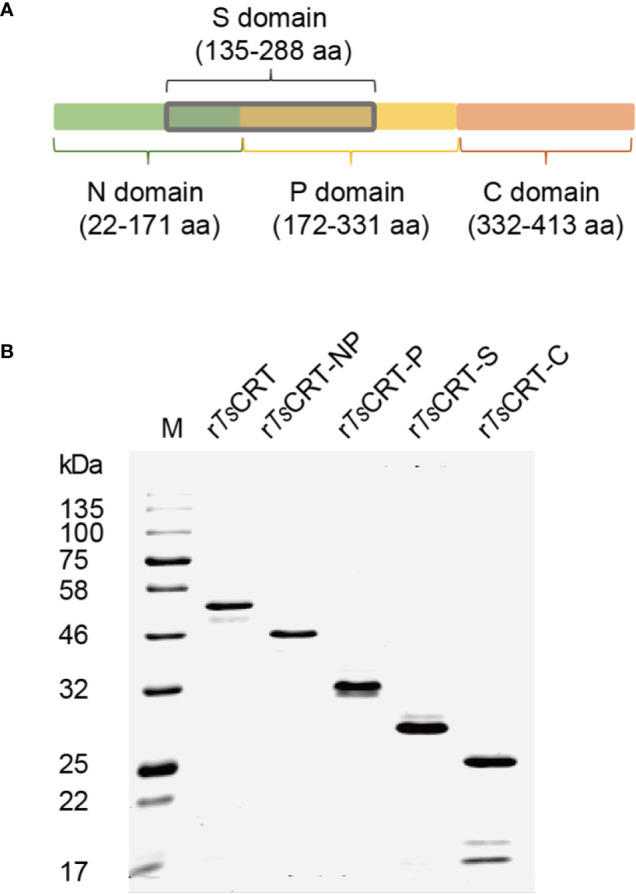
Expression of recombinant *Ts*CRT and its fragment domains. **(A)** *Ts*CRT domain allocation. **(B)** r*Ts*CRT and its fragments (r*Ts*CRT-NP, r*Ts*CRT-P, r*Ts*CRT-S, and r*Ts*CRT-C) were expressed in *E. coli* and analyzed by SDS-PAGE.

To determine the C1q binding sites and protein–protein interactions between C1q and *Ts*CRT, different fragments of *Ts*CRT, including full-length *Ts*CRT (*Ts*CRT, 22–413 aa), the NP-region (*Ts*CRT-NP, 22–331 aa), P-domain (*Ts*CRT-P, 172–331 aa), S-region (*Ts*CRT-S, 135–288 aa), and C-domain (*Ts*CRT-C, 332–413 aa) were expressed as recombinant proteins in *E. coli*. (19) (**Figure 2A**) and the His-tagged soluble recombinant proteins purified using nickel affinity chromatography. The results of SDS-PAGE showed that the purified recombinant full-length and fragment proteins of *Ts*CRT migrated at the predicted molecular weights (r*Ts*CRT 52 kDa, r*Ts*CRT-NP 46 kDa, r*Ts*CRT-P 32 kDa, r*Ts*CRT-S 27 kDa, and r*Ts*CRT-C 25 kDa) (**Figure 2B**). Endotoxin levels in the purified recombinant proteins were < 10 EU/mg.

### *Ts*CRT-S Binds to Human C1q

**Figure 3:**
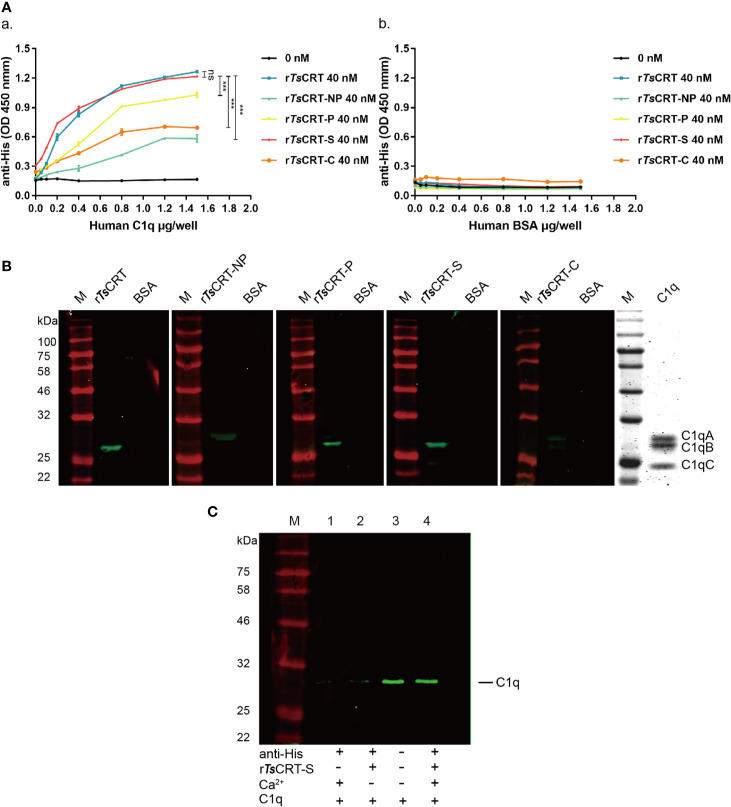
Human C1q binding capacity of different *Ts*CRT fragments. **(A)** ELISA measurement of the binding capacities of different r*Ts*CRT fragments to plates coated with human C1q, detected using anti-His antibody (a). r*Ts*CRT and its fragments did not bind to the BSA control (b). Experiments were repeated three times. Data are shown as the mean ± SEM (***p < 0.001). **(B)** Far western blot showing the binding of C1q with r*Ts*CRT and its fragments. 5 µg of C1q and BSA were transferred to nitrocellulose membranes, then incubated with 5 µg/ml r*Ts*CRT, r*Ts*CRT-NP, r*Ts*CRT-P, r*Ts*CRT-S, and r*Ts*CRT-C and probed with anti-His antibody (1:5,000). C1q was separated by gel electrophoresis to assess the relative molecular weights of the A, B, and C chains. **(C)** Ca^2+^-dependent r*Ts*CRT-S binding to C1q. C1q was pulled down by immunoprecipitation using r*Ts*CRT-S bound on anti-His IgG/ProteinG beads in the buffer with and without Ca^2+^. Eluted complexes were separated by SDS-PAGE and transferred to a nitrocellulose membrane and then detected using a rabbit anti-C1qA antibody. M, molecular weight marker.

The binding of different fragments of *Ts*CRT with C1q was confirmed using different immunological assays. ELISAs using C1q-coated plates demonstrated that r*Ts*CRT, r*Ts*CRT-NP, r*Ts*CRT-P, r*Ts*CRT-S, and r*Ts*CRT-C all bound to C1q in a C1q dose-dependent manner. When plates were coated with 0.8 µg/well of C1q, almost all fragments reached saturated binding at a protein concentration of 40 nM. Among the fragments, r*Ts*CRT-S showed the highest C1q binding capacity, with a similar level to that of r*Ts*CRT and significantly higher binding capacity than r*Ts*CRT-NP, r*Ts*CRT-P, and r*Ts*CRT-C (*p* < 0.001) (**Figure 3Aa**). There was no obvious binding of any *Ts*CRT fragment to BSA under the same conditions, indicating that r*Ts*CRT fragments bound specifically to C1q (**Figure 3Ab**).

Far western blotting with C1q transferred on to a membrane and incubated with different fragments of *Ts*CRT demonstrated that r*Ts*CRT, r*Ts*CRT-P, and r*Ts*CRT-S bound to C1qB, while r*Ts*CRT-NP mostly bound to C1qA, and r*Ts*CRT-C bound to C1qA and B, as determined by detection using anti-His antibody. From the binding density more C1q appeared to be bound to r*Ts*CRT-S and r*Ts*CRT than to r*Ts*CRT-P, r*Ts*CRT-NP, and r*Ts*CRT-C. There was no C1q bound to the BSA control (**Figure 3B**). Interestingly, C1q could not bind to denatured recombinant *Ts*CRT fragments transferred onto a membrane, indicating that C1q only binds to recombinant *Ts*CRT fragments with the correct tertiary confirmation (data not shown).

To confirm whether binding of r*Ts*CRT-S to C1q requires Ca^2+^, an immunoprecipitation assay was performed to pull down C1q using r*Ts*CRT-S and anti-His IgG immobilized on ProteinG MicroBeads under non-denaturing conditions. C1q was only efficiently pulled down by r*Ts*CRT-S immobilized on beads in reaction buffer containing Ca^2+^. In the absence of Ca^2+^ in the buffer, C1q was barely detectable on the stripped beads, similar to the reaction without r*Ts*CRT-S (**Figure 3C**). These results confirm that r*Ts*CRT-S has similar C1q binding ability to r*Ts*CRT and that the binding reaction requires the native conformation and is Ca^2+^ dependent.

### *Ts*CRT-S Inhibits the Classical Complement Activation Pathway Through Binding to C1q 

**Figure 4:**
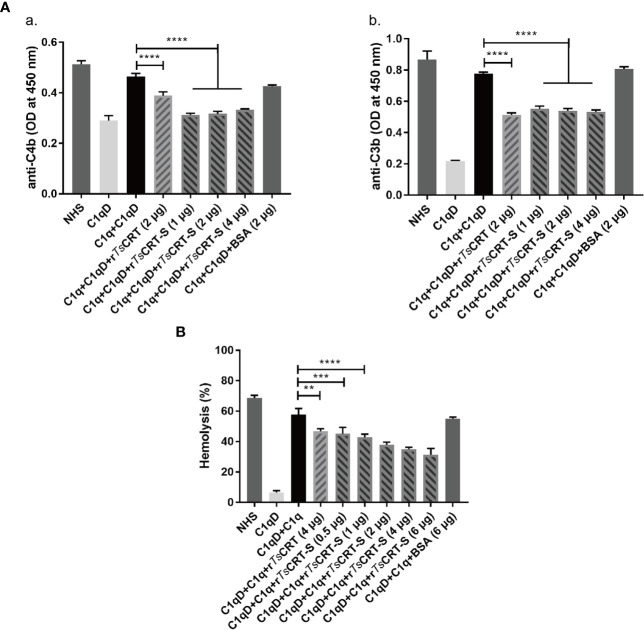
Inhibition of classical complement activation by r*Ts*CRT-S binding to C1q. **(A)** Inhibition of IgM-C1q initiated activation of the intermediary products of classical complement activation, C4b/C3b, as detected by ELISA. C1q (1 µg) was pre-incubated with r*Ts*CRT-S (0, 1, 2, or 4 µg), r*Ts*CRT (2 µg), or BSA (2 µg), then transferred into a 96-well plate coated with human IgM (2 µg/ml). C1q-D serum was supplemented to trigger the IgM-C1q-activated classical pathway, and the generated C4b and C3b detected by anti-C4b and anti-C3b antibodies (1:5,000). **(B)** Sheep red blood cell (SRBC) hemolysis was inhibited in a dose-dependent manner when C1q (1 µg) was incubated with different amounts of r*Ts*CRT-S (0, 0.5, 1, 2, 4, or 6 µg). Each experiment was repeated three times. Data are shown as the mean ± SEM (**p < 0.01, ***p < 0.001, and ****p < 0.0001).

To determine the ability of r*Ts*CRT-S to inhibit C1q-initiated classical complement activation, the IgM-initiated C1q activation intermediate products, C4b and C3b, were measured using an ELISA assay. When 4 µg C1q was pre-incubated with different amounts of r*Ts*CRT-S, similar significant reductions in C4b and C3b products were observed in the reaction when 1 µg of r*Ts*CRT-S or 2 µg of r*Ts*CRT was added (**Figure 4A**).

The hemolysis caused by C1q-initiated classical complement activation was also significantly reduced by addition of r*Ts*CRT-S. As shown in **Figure 4B**, approximately 60% hemolysis occurred when C1q and C1q-D serum were added to sheep red blood cells (SRBCs) sensitized with rabbit anti-SRBC antibody; however, when C1q was pre-incubated with r*Ts*CRT-S, hemolysis was significantly reduced in a dose-dependent fashion. r*Ts*CRT-S as low as 0.5 µg inhibited hemolysis at a similar level to 4 µg r*Ts*CRT. There was no inhibitory effect when BSA control protein was used. These results indicate that r*Ts*CRT-S has the same, or even stronger, ability as r*Ts*CRT to inhibit the activation of the classical complement pathway by binding to C1q.

### r*Ts*CRT-S Inhibits C1q Binding to Neutrophil-Like Cells

**Figure 5:**
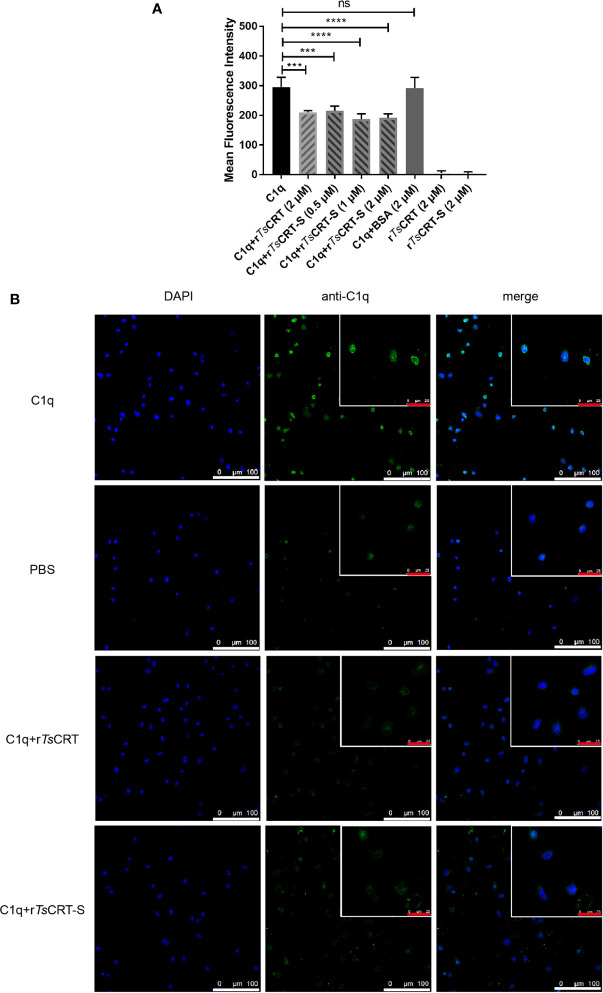
r*Ts*CRT-S inhibited C1q binding to neutrophils. **(A)** C1q binding to neutrophils was inhibited by r*Ts*CRT-S, as detected by high-content screening. ATRA-stimulated dHL60 cells were incubated with C1q (25 nM) that had been pre-incubated with r*Ts*CRT-S (0, 0.5, 1, and 2 µM), r*Ts*CRT (2 µM), or BSA (2 µM). Binding of C1q on dHL60 cells was detected by anti-C1q mAb and DyLight 488-conjugated anti-Rat IgG (green). The experiment was repeated three times. Data are shown as the mean ± SEM (***p < 0.001, and ****p < 0.0001; ns = no significant difference). **(B)** Inhibition of C1q binding to dHL60 cells by r*Ts*CRT-S shown by confocal photography. Binding of C1q (green) on dHL60 cells is visible at the edge of the cells. Nuclei were stained with DAPI (blue). The white scale bars of the photographs represent 100 µm, and the red scale bars of the cells at the top right corner represent 25 µm.

HL60 cells were treated with 1 µM ATRA for 5 days to induce differentiation into neutrophil-like cells (34), which was confirmed by the expression of CD11b and CD66a on their surface (data not shown). To investigate whether r*Ts*CRT-S, as well as r*Ts*CRT, could inhibit C1q binding to the C1q receptor on dHL60 cells, C1q was pre-incubated with various concentrations of r*Ts*CRT-S (0, 0.5, 1.0, and 2.0 µM) or 2 µM r*Ts*CRT. C1q binding on dHL60 was effectively inhibited by pre-incubation with r*Ts*CRT-S or r*Ts*CRT. Fluorescence density, measured by High Content Screening, showed that incubation with r*Ts*CRT-S significantly inhibited C1q binding on dHL60 and that 0.5 µM r*Ts*CRT-S induced inhibition at a similar level to that induced by 2 µM r*Ts*CRT (**Figure 5A**). Treatment with r*Ts*CRT-S or r*Ts*-CRT alone, without C1q, yielded no obvious ﬂuorescence detection on cells. This inhibition was also detected by immunofluorescence using an anti-C1q antibody (**Figure 5B**). These results indicate that the binding of r*Ts*CRT-S or r*Ts*CRT to C1q can inhibit C1q binding to the C1q receptor on neutrophil-like dHL60 cells, possibly through competitive binding to C1q sites that bind to dHL60.

### r*Ts*CRT-S Inhibits C1q-Induced Neutrophil Activity

**Figure 6:**
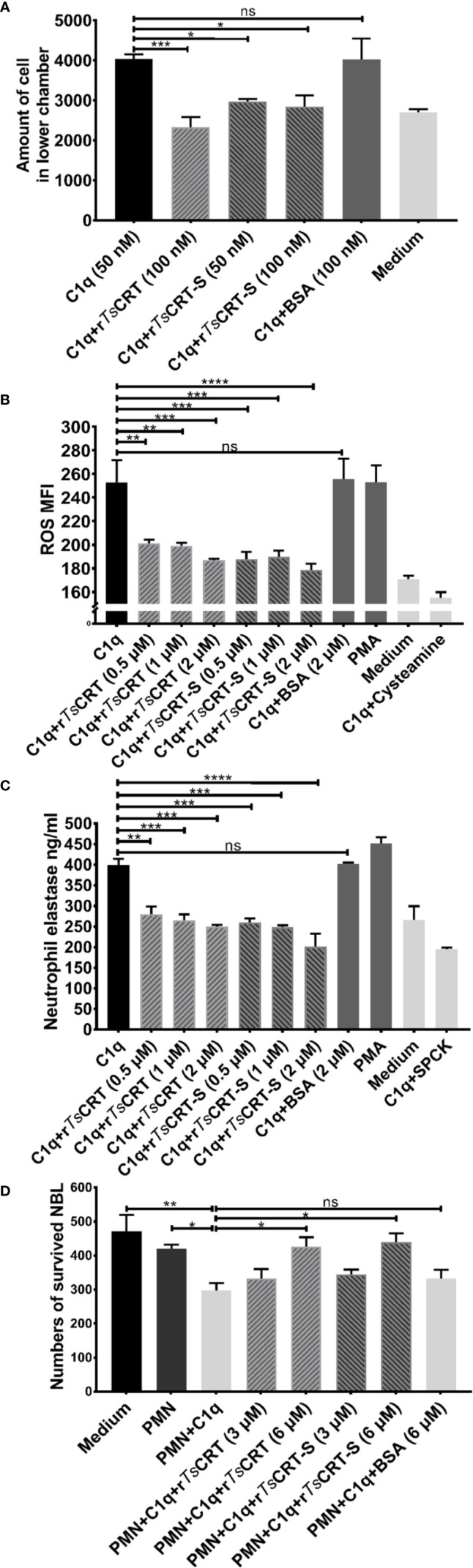
r*Ts*CRT-S inhibited C1q-triggered neutrophil chemotaxis and function. **(A)** C1q-induced neutrophil chemotaxis was inhibited by r*Ts*CRT-S in a transwell migration chamber. The number of cells traversing the membrane was counted. **(B)** r*Ts*CRT-S inhibited dHL60 cell release of ROS and **(C)** neutrophil elastase (NE) through binding to C1q. DCFH-DA was used to probe ROS production, and NE substrate was used to evaluate NE production, which was also quantified as MFI. SPCK was used as an NE inhibitor and cysteamine used as a ROS inhibitor. **(D)** C1q-mediated neutrophil killing of NBL was inhibited by r*Ts*CRT-S. ATP activity was measured as a marker for NBL viability. ATP levels were calculated using a standard curve generated from the ATP levels in untreated control NBL. Each experiment was repeated three times. Data are shown as the mean ± SEM (*p < 0.05, **p < 0.01, ***p < 0.001, and ****p < 0.0001; ns = no significant difference).

To determine whether C1q-induced neutrophil chemotaxis could be suppressed by r*Ts*CRT-S binding, a transwell migration assay was conducted. The results showed that C1q alone attracted dHL60 migration through the inserted membrane; however, C1q-induced migration was significantly inhibited after C1q was incubated with different concentrations of r*Ts*CRT-S (**Figure 6A**). r*Ts*CRT also significantly inhibited C1q-induced neutrophil cell migration. BSA protein did not affect dHL60 cells attraction by C1q.

C1q induced neutrophil secretion of ROS and NE was measured to determine whether r*Ts*CRT-S or r*Ts*CRT interfered with C1q-triggered release of bioactive products by neutrophils. C1q alone induced significant release of ROS and NE, which act as defense mechanisms to kill invading pathogens (39); however, binding with r*Ts*CRT-S or r*Ts*CRT significantly inhibited C1q stimulation of dHL60 cell release of ROS (**Figure 6B**) and NE (**Figure 6C**). The inhibitory effect was highest when 2 µM r*Ts*CRT-S or r*Ts*CRT was added. No obvious inhibition was detected following incubation of C1q with BSA (2 µM), whereas incubation of the cells with the ROS inhibitor, cysteamine, or the NE inhibitor, SPCK, significantly reduced the detectable levels of ROS and NE, respectively. PMA was used as positive control to stimulate neutrophil release of ROS and NE.

To investigate the ability of C1q-induced neutrophils to attack NBL, an ATP-based larval viability assay was established. As shown in **Figure 6D**, C1q could directly induce PMN cells to attack and reduce the viability of NBL; however, when C1q was pre-incubated with r*Ts*CRT-S or r*Ts*CRT, C1q-induced PMN-mediated NBL killing was significantly reduced. At 6 µM r*Ts*CRT-S or r*Ts*CRT, C1q-induced PMN killing of NBL was completely inhibited. BSA had no effect on the ability of C1q to induce PMN killing of NBL.

These results indicate that r*Ts*CRT-S has similar effects to r*Ts*CRT in inhibiting C1q-induced neutrophil functions, including chemotaxis, release of bioactive products, and worm killing capacity.

### r*Ts*CRT-S Inhibits IC-C1q-Induced NET Formation

**Figure 7:**
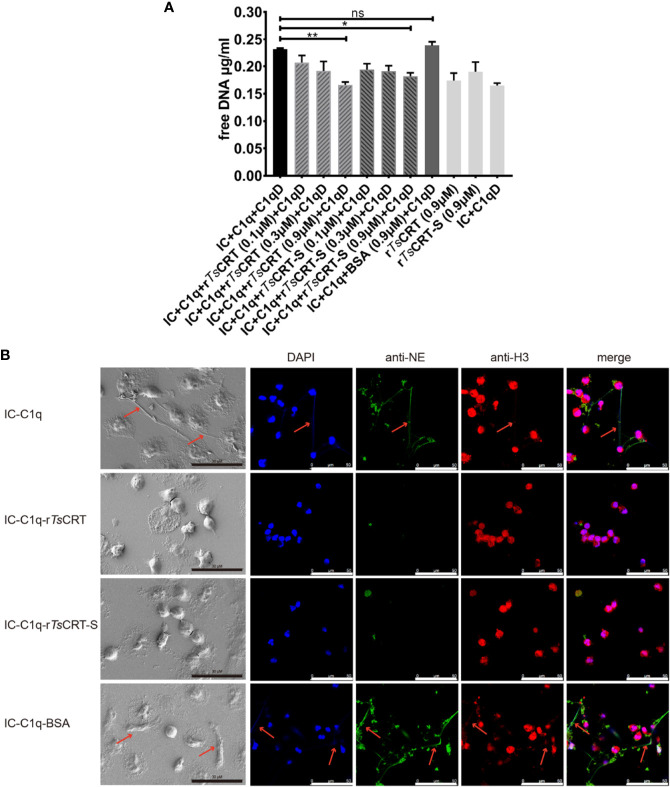
r*Ts*CRT-S inhibits human PMN formation of neutrophil extracellular traps (NETs) through inhibiting C1q-initiated classical complement activation. **(A)** PMN release of free DNA was measured on treatment with C1q (2 µg) pre-incubated with r*Ts*CRT-S or r*Ts*CRT (0, 0.1, 0.3, and 0.9 µM) or BSA (0.9 µM). C1q-D serum and IC were added to activate the classical complement pathway. The free DNA experiment was repeated three times. Data are shown as means ± SEM (*p < 0.05, **p < 0.01; ns = no significant difference). **(B)** NETosis was visualized using scanning electron microscopy (SEM) and confocal immune fluorescence microscopy (IF). Reduced NETosis, and PMN release of NE and histone H3, were observed when C1q was incubated with r*Ts*CRT-S and r*Ts*CRT, but not the BSA control. The black scale bars of the SEM represent 30 µm, and the white scale bars of the IF represent 50 µm. Arrowheads indicate the NET formation.

To evaluate the effect of r*Ts*CRT-S on NET formation induced by the C1q-initiated classical complement pathway activation, IC (albumin/anti-albumin complex) was used to trigger C1q-induced activation. IC-initiated C1q-dependent complement activation significantly induced PMN to form NET; however, when C1q was pre-incubated with r*Ts*CRT-S, C1q-initiated NETosis was significantly reduced, as characterized by the reduction in free DNA (**Figure 7A**), reduction of extracellular fiber network formation, and reduction in levels of elastase and histone H3 released from the network fibers (**Figure 7B**). r*Ts*CRT-S dose-dependently inhibited the release of free DNA, with the highest inhibition achieved with 0.9 µM r*Ts*CRT-S, similar to the effect of r*Ts*CRT. NETosis was also reduced in dHL60 cells when C1q-initiated complement activation was inhibited by r*Ts*CRT-S binding (data not shown). These findings demonstrate that IC-C1q-initiated complement activation stimulates NETosis of PMN or neutrophil-like dHL60 cells and can be inhibited by r*Ts*CRT-S through binding to C1q.

## Discussion

Helminths have developed sophisticated mechanisms to escape host immune attack, particularly by producing molecules to modulate host immune responses, as a survival strategy. Complement is the first line of innate immunity in the defense against pathogen invasion, including with parasitic infections. Activated complement components not only directly attack pathogens by forming a membrane attack complex but also enhance the abilities of antibodies and effector cells (neutrophils, macrophages, and eosinophils, *etc*.) to clear microbes and damaged cells from the infected organism (7). Calreticulin is one such protein secreted by parasitic helminths to evade complement-mediated immune attack (13, 21, 40). The C1 complex, comprising C1q–C1s2–C1r2, is the initiator of the classical complement pathway. C1q is the major binding and effective target of helminth calreticulin in its interference with C1q-initiated classical complement activation, as well as C1q-mediated immune cell functions (4, 8).

In our previous study, we characterized *T. spiralis* calreticulin as a strong C1q binding protein that can inhibit the classical complement pathway and macrophage function (5). To determine the C1q binding site in *Ts*CRT, the 3D structure of *Ts*CRT was predicted, using the crystal structure of human calreticulin arm domains (PDB: 3RG0) as a template.

Molecular docking of *Ts*CRT with human complement C1q showed that amino acids in the C1q head region (particularly ghB and ghC) mainly interacted with the N-domain and P-domain of *Ts*CRT. In general, hydrophobic, electrostatic interactions and hydrogen bonds were formed among *Ts*CRT amino acids and HuC1q. It is also noteworthy that those amino acid residues (Arg^B108^, Arg^B109^, Arg^B114^, His^B117^, Arg^C156^) on C1q involved in the C1q–*Ts*CRT interaction are also involved in C1q–IgG, C1q–IgM, and C1q–gC1qR interactions, indicating that *Ts*CRT binding to C1q could competitively block C1q binding to the IC, consequently inhibiting IC–C1q-initiated classical complement activation and other immune cell responses (**Table 2**).

As shown by the protein-protein docking model, r*Ts*CRT-S, which spans the N and P domains, exhibits C1q binding capacity, indicating its importance in the interaction of *Ts*CRT with C1q. This is in accordance with previous publications describing that the C1q binding site in calreticulin was mainly localized to the S-domain (11, 19). Based on this analysis, the *Ts*CRT domain fragments, including the S-domain, were expressed as recombinant proteins in *E. coli*. Binding assays with different *Ts*CRT fragments confirmed that r*Ts*CRT-S has C1q binding capacity similar to that of full-length r*Ts*CRT, and significantly stronger than those of the other fragments (r*Ts*CRT-NP, r*Ts*CRT-P, and r*Ts*CRT-C). We also demonstrate that C1q binding ability of r*Ts*CRT-S is dependent on the tertiary protein conformation and Ca^2+^ dependent. r*Ts*CRT-S could not bind to C1q under denaturing conditions or in the absence of Ca^2+^. Hence, our results show that Ca^2+^ binding is not only an intrinsic property of CRT, but also necessary for C1q binding with its ligands that is in accordance with the previous study (37). Far western blotting with C1q transferred onto a membrane and bound to different fragments of *Ts*CRT confirmed that r*Ts*CRT-S bound strongly to C1qB, similar to full-length *Ts*CRT. Surprisingly, r*Ts*CRT-NP primarily bound to C1qA, while r*Ts*CRT-C bound to both C1qA and C1qB. Although *Ts*CRT-S is located within *Ts*CRT-NP, the latter showed lower C1q binding capacity and bound to a different part of the C1q complex C1qA, while r*Ts*CRT-S bound to C1qB. It is possible that r*Ts*CRT-NP exhibits a different tertiary conformational structure compared with r*Ts*CRT-S.

Our results confirm that the C1q binding site in *Ts*CRT is located in *Ts*CRT-S. Further, our study demonstrates that r*Ts*CRT-S shared a comparable capacity with full-length r*Ts*CRT to interfere with human C1q functions through C1q binding. After binding to C1q, r*Ts*CRT-S significantly inhibited the IgM–C1q-initiated classical complement activation pathway, characterized by a reduction in intermediate products (C4b and C3b) and reduced sheep red blood cell hemolysis.

In addition to inhibiting the C1q-initiated classical complement pathway, r*Ts*CRT-S also inhibited C1q binding to its ligands on neutrophils and their consequent functions. Neutrophils express C1q receptor on their surface and C1q induces neutrophil chemotaxis to inflammatory sites and stimulates neutrophil degranulation and production of oxidative and effector products as an immune response to invaded pathogens (14, 15). Surface gC1qR binds to the globular heads of C1q and enhances the chemotactic potency of C1q (14). In this study, we demonstrate that, after being bound with r*Ts*CRT-S, binding of C1q to the surface of ATRA-stimulated dHL60 (neutrophil-like) cells was inhibited, possibly through competitive binding to the C1q receptor on dHL60 cells. Binding with r*Ts*CRT-S not only significantly reduced the chemotaxis effects of C1q on neutrophils (dHL60 cells), as detected by transwell chamber assays, but also significantly reduced dHL60 cells release of NE and ROS.

Neutrophils are often the first cells to be recruited to the site of invaded pathogens, such as parasitic helminths. ROS and granular proteins (NE, *etc*.) are directly involved in killing and clearing infections (41, 42). In early studies to determine the immune mechanisms involved in the expulsion of *T. spiralis* infection, the oxidative products and granular proteins released by neutrophils were identified as toxic to NBL (43, 44). C1q can trigger neutrophil release of toxic ROS and granular proteins by a unique CD18-dependent mechanism (15). Here, we determined that binding with r*Ts*CRT-S significantly reduced the function of C1q, thereby decreasing neutrophil release of ROS and NE. When *T. spiralis* NBL were incubated with PMN isolated from human peripheral blood in the presence of C1q for 48 h, 30% of them died; however after binding with 6 µM r*Ts*CRT-S, C1q induced PMN killing of NBL was completed inhibited, indicating that r*Ts*CRT-S can completely inhibit C1q-induced neutrophil killing of NBL in the same way as full-length r*Ts*CRT, possibly through inhibiting C1q-triggered release of oxidative ROS and NE by neutrophils. Furthermore, eosinophils are also important effector cells involved in the protective immunity against helminth infection, mostly associated with the antibody-dependent cell-mediated cytotoxicity (ADCC) (45). Even though there is no evidence that calreticulin is directly involved in the modification of eosinophil functions, it is worth to explore the possible interference of *Ts*CRT with eosinophils since the C1q receptor is also expressed on eosinophils (46).

In addition to directly releasing anti-microbials (such as ROS and NE) to clear the invaded pathogens, neutrophils also secrete chromatin and granule proteins to form extracellular fibril matrices, NET, which were first described as a strategy by which neutrophils capture and kill extracellular pathogens (24). C1q-initiated classical complement activation is involved in NETosis (26). In this study we demonstrate that NETosis is significantly reduced when C1q is pre-incubated with r*Ts*CRT-S, and IC-C1q-iniated classical complement activation was inhibited, indicating that *T. spiralis* secrets *Ts*CRT to inhibit C1q-induced NETosis as another approach to escape immune attack. In addition to their immune-protective function, NET are involved in a number of diseases with immunological pathology (25). An increasing number of clinical cases show that excessive NET accumulation is associated with atherosclerosis, tumor metastasis, and autoimmune diseases (systemic lupus erythematosus, rheumatoid arthritis, etc.) (25, 29). Therefore, inhibition of NETosis has been targeted to develop therapeutics aimed at mitigating excessive complement activation-induced and NET-related autoimmune diseases (47). Due to its specific inhibition of NETosis through inhibiting C1q-induced complement activation, r*Ts*CRT-S has potential for use as a therapeutic agent to ease NET-related autoimmune diseases. Further, because of their strong immunomodulatory functions, helminth infections and helminth-derived proteins have been successfully used to treat a variety of allergies, autoimmune diseases, and other immune dysregulation disorders, such as type 1 diabetes, systemic lupus erythematosus, rheumatoid arthritis, and inflammatory bowel disease (48–52). Our previous studies have demonstrated that *T. spiralis* infection significantly mitigated collagen-induced arthritis via PD-1 mediated immunomodulation (49), that *T. spiralis*-derived excretory/secretory products ameliorate inflammatory colitis in experimental mouse models (53), and that *T. spiralis* adult extracts have preventive and therapeutic effects on asthma inflammation (54). Since *Ts*CRT is secreted by *T. spiralis* as an immunomodulatory protein to neutralize C1q-induced complement activation as an immune escape strategy, there is potential that *Ts*CRT-S could be developed as a therapeutic agent for complement-involved autoimmune diseases.

Overall, we have determined that the dominant C1q binding site in *Ts*CRT is located within *Ts*CRT-S. The 153 amino acid *Ts*CRT-S functional domain has the same capacity as full-length *Ts*CRT to bind to C1q, inhibit C1q-induced complement activation, and interfere with other C1q-induced immune cell activation and functions. The interaction between *Ts*CRT-S and C1q not only directly inhibits membrane damage of parasites caused by C1q-iniatiated classic complement activation, but also inhibits C1q-induced neutrophil parasite killing. The findings of this study further elucidate the immune evasion mechanism of *T. spiralis*, which warrants future study as a target for development of therapeutic drugs or preventive vaccines against trichinellosis. As IC-C1q-induced formation of NET was effectively inhibited by *Ts*CRT-S, there is potential for development of *Ts*CRT-S as therapeutic agent to inhibit NET-involved pathologies, such as autoimmune diseases. Since *Ts*CRT-S is a small functional domain, which confers the full complement of modulatory functions of full-length *Ts*CRT, it will be easier to develop this functional domain as vaccine or therapeutic target, rather than the full-length protein.

## Conclusion

We found that *Ts*CRT binds human complement C1q is mediated by its S-domain. Molecular docking modeling and fragment expression revealed that *Ts*CRT-S is responsible for C1q binding. Similar to the full length of *Ts*CRT, r*Ts*CRT-S expressed in *Escherichia coli* showed the same capacity to bind and therefore inhibit human C1q-induced complement activation. The interaction could also inhibit release of reactive oxygen species and elastase by neutrophil when triggered by C1q, resulting in reduced neutrophil killing of NBL. Lastly, binding of *Ts*CRT-S to C1q also inhibited formation of neutrophil extracellular traps (NET) indicating a potential therapeutical application in treating autoimmune diseases.

## Data Availability Statement

The original contributions presented in the study are included in the article/supplementary materials. Further inquiries can be directed to the corresponding author.

## Ethics Statement

The studies involving human participants were reviewed and approved by Institutional Review Board of Capital Medical University (approval number: 2016SY01). The ethics committee waived the requirement of written informed consent for participation. The animal study was reviewed and approved by Capital Medical University Animal Care and Use Committee (approval number: AEEI-2017-133).

## Author Contributions

XZ and SS conceived and designed the experiments. SS, CH, QZ, LZ, YC, and JH performed the experiments. SS, BZ, and XZ analyzed the data. SS, BZ, and XZ drafted the paper. XZ, BZ, CH, LZ, YC, and JH revised the paper. All authors agreed to be accountable for all aspects of the work in ensuring that questions related to the accuracy or integrity of any part of the work are appropriately investigated and resolved. All authors contributed to the article and approved the submitted version.

## Funding

This work was supported by a grant from the National Natural Science Foundation of China (81672042).

## Conflict of Interest

The authors declare that the research was conducted in the absence of any commercial or financial relationships that could be construed as a potential conflict of interest.

## References

[1] Dupouy-Camet J Trichinellosis: a worldwide zoonosis. Vet Parasitol (2000) 93(3-4):191–200. 10.1016/s0304-4017(00)00341-1 11099837

[2] Gottstein B Pozio E Nockler K Epidemiology, diagnosis, treatment, and control of trichinellosis. Clin Microbiol Rev (2009) 22(1):127–45. 10.1128/cmr.00026-08. PMC2620635 19136437

[3] Leid RW Parasites and complement. Adv Parasitol (1988) 27:131–68. 10.1016/s0065-308x(08)60354-1 3289328

[4] Shao S Sun X Chen Y Zhan B Zhu X Complement Evasion: An Effective Strategy That Parasites Utilize to Survive in the Host. Front Microbiol (2019) 10:532. 10.3389/fmicb.2019.00532 30949145 PMC6435963

[5] Zhao L Shao S Chen Y Sun X Sun R Huang J Trichinella spiralis Calreticulin Binds Human Complement C1q As an Immune Evasion Strategy. Front Immunol (2017) 8:636. 10.3389/fimmu.2017.00636 28620388 PMC5449505

[6] Kishore U Reid KB C1q: structure, function, and receptors. Immunopharmacology (2000) 49(1-2):159–70. 10.1016/s0162-3109(00)80301-x 10904115

[7] Nayak A Ferluga J Tsolaki AG Kishore U The non-classical functions of the classical complement pathway recognition subcomponent C1q. Immunol Lett (2010) 131(2):139–50. 10.1016/j.imlet.2010.03.012 20381531

[8] Thielens NM Tedesco F Bohlson SS Gaboriaud C Tenner AJ C1q: A fresh look upon an old molecule. Mol Immunol (2017) 89:73–83. 10.1016/j.molimm.2017.05.025 28601358 PMC5582005

[9] Ramirez-Toloza G Ferreira A Trypanosoma cruzi Evades the Complement System as an Efficient Strategy to Survive in the Mammalian Host: The Specific Roles of Host/Parasite Molecules and Trypanosoma cruzi Calreticulin. Front Microbiol (2017) 8:1667. 10.3389/fmicb.2017.01667 28919885 PMC5585158

[10] Ramirez-Toloza G Sosoniuk-Roche E Valck C Aguilar-Guzman L Ferreira VP Ferreira A Trypanosoma cruzi Calreticulin: Immune Evasion, Infectivity, and Tumorigenesis. Trends Parasitol (2020) 36(4):368–81. 10.1016/j.pt.2020.01.007 32191851

[11] Yadav S Gupta S Selvaraj C Doharey PK Verma A Singh SK In silico and in vitro studies on the protein-protein interactions between Brugia malayi immunomodulatory protein calreticulin and human C1q. PloS One (2014) 9(9):e106413. 10.1371/journal.pone.0106413 25184227 PMC4153637

[12] Suchitra S Joshi P Characterization of Haemonchus contortus calreticulin suggests its role in feeding and immune evasion by the parasite. Biochim Biophys Acta (2005) 1722(3):293–303. 10.1016/j.bbagen.2004.12.020 15716049

[13] Kasper G Brown A Eberl M Vallar L Kieffer N Berry C A calreticulin-like molecule from the human hookworm Necator americanus interacts with C1q and the cytoplasmic signalling domains of some integrins. Parasite Immunol (2001) 23(3):141–52. 10.1046/j.1365-3024.2001.00366.x 11240905

[14] Leigh LE Ghebrehiwet B Perera TP Bird IN Strong P Kishore U C1q-mediated chemotaxis by human neutrophils: involvement of gClqR and G-protein signalling mechanisms. Biochem J (1998) 330(Pt 1):247–54. 10.1042/bj3300247 PMC1219134 9461517

[15] Goodman EB Anderson DC Tenner AJ C1q triggers neutrophil superoxide production by a unique CD18-dependent mechanism. J leukocyte Biol (1995) 58(2):168–76. 10.1002/jlb.58.2.168 7643012

[16] Kolaczkowska E Kubes P Neutrophil recruitment and function in health and inflammation. Nat Rev Immunol (2013) 13(3):159–75. 10.1038/nri3399 23435331

[17] Ghai R Waters P Roumenina LT Gadjeva M Kojouharova MS Reid KB C1q and its growing family. Immunobiology (2007) 212(4-5):253–66. 10.1016/j.imbio.2006.11.001 17544811

[18] Michalak M Groenendyk J Szabo E Gold LI Opas M Calreticulin, a multi-process calcium-buffering chaperone of the endoplasmic reticulum. Biochem J (2009) 417(3):651–66. 10.1042/bj20081847 19133842

[19] Stuart GR Lynch NJ Lu J Geick A Moffatt BE Sim RB Localisation of the C1q binding site within C1q receptor/calreticulin. FEBS Lett (1996) 397(2-3):245–9. 10.1016/s0014-5793(96)01156-8 8955356

[20] Stuart GR Lynch NJ Day AJ Schwaeble WJ Sim RB The C1q and collectin binding site within C1q receptor (cell surface calreticulin). Immunopharmacology (1997) 38(1-2):73–80. 10.1016/s0162-3109(97)00076-3 9476117

[21] Naresha S Suryawanshi A Agarwal M Singh BP Joshi P Mapping the complement C1q binding site in Haemonchus contortus calreticulin. Mol Biochem Parasitol (2009) 166(1):42–6. 10.1016/j.molbiopara.2009.02.007 19428671

[22] Holers VM Complement and Its Receptors: New Insights into Human Disease. Annu Rev Immunol (2014) 32(1):433–59. 10.1146/annurev-immunol-032713-120154 24499275

[23] Leffler J Bengtsson AA Blom AM The complement system in systemic lupus erythematosus: an update. Ann Rheum Dis (2014) 73(9):1601–6. 10.1136/annrheumdis-2014-205287 24845390

[24] Sollberger G Tilley DO Zychlinsky A Neutrophil Extracellular Traps: The Biology of Chromatin Externalization. Dev Cell (2018) 44(5):542–53. 10.1016/j.devcel.2018.01.019 29533770

[25] Papayannopoulos V Neutrophil extracellular traps in immunity and disease. Nat Rev Immunol (2018) 18(2):134–47. 10.1038/nri.2017.105 28990587

[26] de Bont CM Boelens WC Pruijn GJM NETosis, complement, and coagulation: a triangular relationship. Cell Mol Immunol (2019) 16(1):19–27. 10.1038/s41423-018-0024-0 29572545 PMC6318284

[27] Sorensen OE Borregaard N Neutrophil extracellular traps - the dark side of neutrophils. J Clin Invest (2016) 126(5):1612–20. 10.1172/jci84538 PMC4855925 27135878

[28] Jorch SK Kubes P An emerging role for neutrophil extracellular traps in noninfectious disease. Nat Med (2017) 23(3):279–87. 10.1038/nm.4294 28267716

[29] Lee KH Kronbichler A Park DD Park Y Moon H Kim H Neutrophil extracellular traps (NETs) in autoimmune diseases: A comprehensive review. Autoimmun Rev (2017) 16(11):1160–73. 10.1016/j.autrev.2017.09.012 28899799

[30] Trouw LA Pickering MC Blom AM The complement system as a potential therapeutic target in rheumatic disease. Nat Rev Rheumatol (2017) 13(9):538–47. 10.1038/nrrheum.2017.125 28794515

[31] Rincón E Rocha-Gregg BL Collins SR A map of gene expression in neutrophil-like cell lines. BMC genomics (2018) 19(1):573. 10.1186/s12864-018-4957-6 PMC6090850 30068296

[32] Verstuyf A Mathieu C Verlinden L Waer M Tan BK Bouillon R Differentiation induction of human leukemia cells (HL60) by a combination of 1,25-dihydroxyvitamin D3 and retinoic acid (all trans or 9-cis). J Steroid Biochem Mol Biol (1995) 53(1-6):431–41. 10.1016/0960-0760(95)00089-i 7626492

[33] Almzaiel AJ Billington R Smerdon G Moody AJ Effects of hyperbaric oxygen treatment on antimicrobial function and apoptosis of differentiated HL-60 (neutrophil-like) cells. Life Sci (2013) 93(2-3):125–31. 10.1016/j.lfs.2013.06.003 23770209

[34] Mihara K Nakayama T Saitoh H A Convenient Technique to Fix Suspension Cells on a Coverslip for Microscopy. Curr Protoc Cell Biol (2015) 68:4.30.1–4.10. 10.1002/0471143030.cb0430s68 26331985

[35] Riss TL Moravec RA Niles AL Duellman S Benink HA Worzella TJ Cell Viability Assays. In: Sittampalam GS Grossman A Brimacombe K Arkin M Auld D Austin CP, editors. Assay Guidance Manual. Bethesda (MD: Eli Lilly & Company and the National Center for Advancing Translational Sciences (2004). 23805433

[36] Ishiwata K Watanabe N Nippostrongylus brasiliensis: reversibility of reduced-energy status associated with the course of expulsion from the small intestine in rats. Exp Parasitol (2007) 117(1):80–6. 10.1016/j.exppara.2007.03.019 17482164

[37] Gaboriaud C Juanhuix J Gruez A Lacroix M Darnault C Pignol D The crystal structure of the globular head of complement protein C1q provides a basis for its versatile recognition properties. J Biol Chem (2003) 278(47):46974–82. 10.1074/jbc.M307764200 12960167

[38] Pednekar L Pathan AA Paudyal B Tsolaki AG Kaur A Abozaid SM Analysis of the Interaction between Globular Head Modules of Human C1q and Its Candidate Receptor gC1qR. Front Immunol (2016) 7:567. 10.3389/fimmu.2016.00567 28018340 PMC5153404

[39] Chen Y Junger WG Measurement of oxidative burst in neutrophils. Methods Mol Biol (Clifton NJ) (2012) 844:115–24. 10.1007/978-1-61779-527-5_8 PMC4214271 22262438

[40] Mendlovic F Ostoa-Saloma P Solis CF Martinez-Ocana J Flisser A Laclette JP Cloning, characterization, and functional expression of Taenia solium calreticulin. J Parasitol (2004) 90(4):891–3. 10.1645/ge-3325rn 15357095

[41] Freudenstein-Dan A Gold D Fishelson Z Killing of schistosomes by elastase and hydrogen peroxide: implications for leukocyte-mediated schistosome killing. J Parasitol (2003) 89(6):1129–35. 10.1645/ge-96r 14740899

[42] Rajamanickam A Munisankar S Bhootra Y Dolla CK Nutman TB Babu S Elevated Systemic Levels of Eosinophil, Neutrophil, and Mast Cell Granular Proteins in Strongyloides Stercoralis Infection that Diminish following Treatment. Front Immunol (2018) 9:207. 10.3389/fimmu.2018.00207 29479356 PMC5811458

[43] Bass DA Szejda P Mechanisms of killing of newborn larvae of Trichinella spiralis by neutrophils and eosinophils. Killing by generators of hydrogen peroxide in vitro. J Clin Invest (1979) 64(6):1558–64. 10.1172/jci109616 PMC371308 41002

[44] Venturiello SM Giambartolomei GH Costantino SN Immune killing of newborn Trichinella larvae by human leucocytes. Parasite immunology (1993) 15(10):559–64. 10.1111/pim.1993.15 7877832

[45] Huang L Appleton JA Eosinophils in Helminth Infection: Defenders and Dupes. Trends Parasitol (2016) 32(10):798–807. 10.1016/j.pt.2016.05.004 27262918 PMC5048491

[46] Kuna P Iyer M Peerschke EI Kaplan AP Reid KB Ghebrehiwet B Human C1q induces eosinophil migration. Clin Immunol Immunopathol (1996) 81(1):48–54. 10.1006/clin.1996.0156 8808641

[47] Barnado A Crofford LJ Oates JC At the Bedside: Neutrophil extracellular traps (NETs) as targets for biomarkers and therapies in autoimmune diseases. J leukocyte Biol (2016) 99(2):265–78. 10.1189/jlb.5BT0615-234R PMC6608010 26658004

[48] Wu Z Wang L Tang Y Sun X Parasite-Derived Proteins for the Treatment of Allergies and Autoimmune Diseases. Front Microbiol (2017) 8:2164. 10.3389/fmicb.2017.02164 29163443 PMC5682104

[49] Cheng Y Zhu X Wang X Zhuang Q Huyan X Sun X Trichinella spiralis Infection Mitigates Collagen-Induced Arthritis via Programmed Death 1-Mediated Immunomodulation. Front Immunol (2018) 9:1566. 10.3389/fimmu.2018.01566 30093899 PMC6070611

[50] Lund ME Greer J Dixit A Alvarado R McCauley-Winter P To J A parasite-derived 68-mer peptide ameliorates autoimmune disease in murine models of Type 1 diabetes and multiple sclerosis. Sci Rep (2016) 6:37789. 10.1038/srep37789 27883079 PMC5121616

[51] Segal Y Blank M Shoenfeld Y Tuftsin phosphorylcholine-a novel compound harnessing helminths to fight autoimmunity. Immunol Res (2018) 66(6):637–41. 10.1007/s12026-018-9051-2 30554380

[52] Wang M Wu L Weng R Zheng W Wu Z Lv Z Therapeutic potential of helminths in autoimmune diseases: helminth-derived immune-regulators and immune balance. Parasitol Res (2017) 116(8):2065–74. 10.1007/s00436-017-5544-5 28664463

[53] Yang X Yang Y Wang Y Zhan B Gu Y Cheng Y Excretory/secretory products from Trichinella spiralis adult worms ameliorate DSS-induced colitis in mice. PloS One (2014) 9(5):e96454. 10.1371/journal.pone.0096454 24788117 PMC4008629

[54] Sun S Li H Yuan Y Wang L He W Xie H Preventive and therapeutic effects of Trichinella spiralis adult extracts on allergic inflammation in an experimental asthma mouse model. Parasites Vectors (2019) 12(1):326. 10.1186/s13071-019-3561-1 31253164 PMC6599242

